# Danaparoid sodium-based anticoagulation therapy for portal vein thrombosis in cirrhosis patients

**DOI:** 10.1186/s12876-019-1140-8

**Published:** 2019-12-16

**Authors:** Takehiro Hayashi, Hajime Takatori, Rika Horii, Kouki Nio, Takeshi Terashima, Noriho Iida, Masaaki Kitahara, Tetsuro Shimakami, Kuniaki Arai, Kazuya Kitamura, Kazunori Kawaguchi, Taro Yamashita, Yoshio Sakai, Tatsuya Yamashita, Eishiro Mizukoshi, Masao Honda, Tadashi Toyama, Kenichiro Okumura, Kazuto Kozaka, Shuichi Kaneko

**Affiliations:** 1 Department of Gastroenterology, Kanazawa University Graduate School of Medical Science, Kanazawa, Ishikawa Japan; 2 Department of Gastroenterology, Yawata Medical Center, Komatsu, Ishikawa Japan; 3 Department of Nephrology, Kanazawa University Graduate School of Medical Science, Kanazawa, Ishikawa Japan; 4 Department of Radiology, Kanazawa University Graduate School of Medical Science, Kanazawa, Ishikawa Japan

**Keywords:** Portal vein thrombosis, Liver cirrhosis, Danaparoid sodium, Antithrombin III

## Abstract

**Background:**

Portal vein thrombosis (PVT) is a common complication of cirrhosis. However, in patients with PVT and cirrhosis, there is no clear evidence supporting effective treatment modalities. In this study, we examined the effectiveness and safety of anticoagulation therapy using danaparoid sodium for PVT in patients with cirrhosis.

**Methods:**

This retrospective study assessed 52 cirrhotic patients with PVT treated with danaparoid sodium for 2 weeks between November 2008 and September 2018. The primary outcome measure was the post-treatment status of PVT assessed by reduction in thrombus volume and safety of the therapeutic intervention. PVT status was evaluated with contrast-enhanced computed tomography (CECT). All patients received 1250 units of danaparoid sodium twice daily by intravenous injection for 14 days. Patients on antithrombin III (AT-III) combination therapy were additionally administered 1500 units of AT-III on days 1–5 and days 8–12. Effectiveness was evaluated by CECT from between days 13 and 18. The secondary outcome measure was the prognosis of PVT.

**Results:**

All patients showed reduction in PVT volume without complications. Return of plasma AT-III level to > 70% during the treatment period contributes to ≥75% reduction of PVT volume. The prognosis in PVT patients depends on hepatic reserve capacity. When limited to Child-Pugh B and C liver cirrhosis patients, a ≥ 75% reduction of PVT volume improved the prognosis.

**Conclusions:**

Danaparoid sodium-based anticoagulation therapy was effective and safe for PVT in patients with cirrhosis. Return of plasma AT-III level to the normal range during the treatment period contributes to reduction of PVT volume. A reduction of ≥75% in PVT volume may improve the prognosis of Child-Pugh B and C decompensated cirrhosis patients with PVT.

## Background

Portal vein thrombosis (PVT) is characterized by thrombus formation in the trunk of the portal vein involving its right and left intrahepatic branches. The thrombus may even extend to the splenic or superior mesenteric vein or toward the liver involving the intrahepatic portal branches [1]. PVT may occur in various clinical pathologies, such as cirrhosis, myeloproliferative disease, cancer, and infection [2]. PVT is a common complication of cirrhosis, with prevalence ranging from 0.6 to 15.8% in patients with liver cirrhosis or portal hypertension [3]. Advanced or decompensated cirrhosis and hepatobiliary malignancy are especially high-risk conditions for PVT, and patients with both cirrhosis and hepatic carcinoma are considered to have the highest risk for PVT [4].

PVT has various clinical presentations, ranging from asymptomatic to life-threatening conditions, such as gastroesophageal bleeding and acute intestinal ischemia [3, 5]. Furthermore, PVT is reported to be associated with an increased risk of ascites and mortality risk in patients with cirrhosis [6].

The treatment of PVT should be tailored according to the patient’s background. However, in patients with PVT and cirrhosis, no evidence supports the use of anticoagulation therapy because of the risk of bleeding due to reduced synthesis of coagulation factors and high incidence of varices and portal hypertensive gastropathy [1, 2, 7].

Current treatment modalities for PVT mainly include anticoagulation, systemic and local thrombolysis, percutaneous portal vein recanalization, and transjugular intrahepatic portosystemic shunt [3, 8]. Recent reports have shown that anticoagulation therapy for PVT is safe, with low rates of complication. For example, danaparoid sodium and antithrombin III (AT-III) are effective and safe for treating PVT in patients with liver disease [9–13].

Danaparoid sodium is an anticoagulant that works by inhibiting activated factor Xa. A major advantage of danaparoid is its low rate of cross-reactivity with antibodies associated with immune-mediated heparin-induced thrombocytopenia [14]. Further, it has been reported that danaparoid sodium is unlikely to cause gastrointestinal hemorrhage, and it appears to be more effective and safer than heparin in terms of bleeding complications [15].

In this study, we examined the effectiveness of anticoagulation therapy using danaparoid sodium in PVT in patients with cirrhosis.

## Methods

### Patients

This retrospective study involved 55 cirrhotic patients with PVT treated with danaparoid sodium between November 2008 and September 2018. PVT was defined as the occurrence of thrombosis in the main trunk of the portal vein or the first-order vessels of the left (LPV) or right (RPV) branch of the portal vein or superior mesenteric vein (SMV). We excluded 1 patient with portal vein tumor thrombus and 2 patients for whom plasma AT-III levels were not examined. Finally, 52 patients who received danaparoid sodium-based anticoagulation therapy for 2 weeks as initial treatment were included in the study. All enrolled patients received routine clinical management of PVT at Kanazawa University Hospital. PVT patients were treated with danaparoid sodium with AT-III (combination therapy) or without AT-III (monotherapy). From November 2008 to September 2011, we used combination therapy to treat PVT. From October 2011 to March 2014, we used monotherapy. Also, from April 2014 to September 2018, patients with an AT-III of ≤70% received combination therapy, and those with an AT-III > 70% received monotherapy. Accordingly, 22 and 30 PVT patients were treated with combination therapy or monotherapy, respectively.

Table 1 shows the clinical characteristics of cirrhotic patients with PVT before treatment. In brief, all patients were clinically diagnosed with cirrhosis. We classified hepatocellular carcinoma (HCC) stage based on the UICC TNM classification algorithm for HCC [16]. In this study, cases with no recurrence following curative treatment were designated absence of HCC. We classified esophageal and gastric varices based on the general rules for endoscopic findings of esophagogastric varices [17] (Table 1).

**Table 1:** Baseline characteristics of patients

	*n* = 52
Sex (male / female)	40/12
Mean age (years)	65 ± 9
Etiology (HBV / HCV / HBV + HCV / NBNC)	7/29/1/15
Child-Pugh score (A / B / C)	13/25/14
Hepatocellular carcinoma (absent / present)	31/21
UICC Stage of hepatocellular carcinoma	
Absent (none / after curative treatment)	19/12
Present (Stage I / II / III / IV)	8/12/0/1
Esophageal varices (absent / present)	1/51
Monotherapy / Combination therapy	30/22
White blood cell count (/μL)	3773 ± 1994
Hemoglobin (g/dL)	11.2 ± 1.8
Platelet count (× 10^4^/μL)	9.0 ± 6.4
Albumin (mg/dL)	3.1 ± 0.5
Total bilirubin (mg/dL)	1.6 ± 1.1
Aspartate aminotransferase (IU/L)	46 ± 24
Alanine aminotransferase (IU/L)	32 ± 19
Gamma-glutamyl transpeptidase (IU/L)	47 ± 59
Prothrombin time (%)	64 ± 14
Prothrombin time/International normalized ratio	1.3 ± 0.2
Fibrinogen degradation product (μg/mL)	15.7 ± 17.1
D-dimer (μg/mL)	7.5 ± 8.1
Antithrombin III (%)	58 ± 17

Table 2 shows the characteristics of PVT. In brief, most thromboses (84% of 52 patients) were located completely or partially within the main trunk of the portal vein. The portal vein was completely obstructed by thrombus in 6 cases (11%). Severity of occlusion was classified according to Bauer’s Classification [18]. Treatment was started ≤30 days of diagnosis in 32 patients (62%). The timing of development of PVT was ≤90 days before diagnosis in 14 patients (27%) (Table 2).

**Table 2:** Characteristics of portal vein thrombosis

Site of portal vein thrombosis	*n* = 52	
MPV	11	21%
MPV + intrahepatic branch	17	32%
MPV + SMV	6	11%
MPV + SMV + intrahepatic branch	3	6%
MPV + SV	2	4%
MPV + SV + intrahepatic branch	2	4%
MPV + SMV + SV	2	4%
MPV + SMV + SV + intrahepatic branch	1	2%
SMV	2	4%
SMV + intrahepatic branch	1	2%
RPV	2	4%
LPV	2	4%
LPV + SV	1	2%
Degree of portal vein thrombosis	*n* = 52	
Partial obstruction	46	89%
Complete obstruction	6	11%
Bauer’s Classification	*n* = 52	
Grade I	0	0%
Grade II	15	29%
Grade III	26	50%
Grade IV	11	21%
Period from diagnosis to treatment	*n* = 52	
≤ 30 days	32	62%
> 31 days	20	38%
Period from last test not showing thrombosis to treatment	*n* = 52	
≤ 90 days	14	27%
91–180	12	23%
> 181 days	14	27%
Undetermined	12	23%

*MPV* Main portal vein, *SMV* Superior mesenteric vein, *SV* splenic vein, *RPV* Right portal vein, *LPV* Left portal vein

Regarding events that could cause PVT and occurred ≤90 days prior to diagnosis, PVT was thought to be associated with treatment of HCC in 9 patients (17%) and with variceal events in 7 patients (13%). PVT was associated with infection in 3 patients (6%) and with arterioportal shunt in 1 patient (2%); the cause was unidentifiable in 32 patients (62%) (Table 3).

**Table 3:** Probable cause of PVT

Probable cause	*n* = 52	
Associated with hepatocellular carcinoma	9	17%
RFA	4	
PEIT	1	
IH Chemo	2	
Surgery	1	
Angio CT	1	
Associated with varices	7	13%
Varix rupture	3	
EVL	2	
EIS	1	
BRTO	1	
AP shunt	1	2%
Infection	3	6%
Biliary tract infection	1	
Intestinal infection	1	
Upper respiratory tract infection	1	
Unknown	32	62%

*RFA* Radiofrequency ablation, *PEIT* Percutaneous ethanol injection therapy, *IH* Chemo, Intrahepatic chemotherapy, Angio *CT* Computed tomographic angiography, *EVL* Endoscopic variceal ligation, *EIS* Endoscopic injection sclerotherapy, *BRTO* Balloon-occluded retrograde transvenous obliteration, *AP* shunt Arterioportal shunt

### Protocol for treatment of portal vein thrombosis

**Fig. 1:**
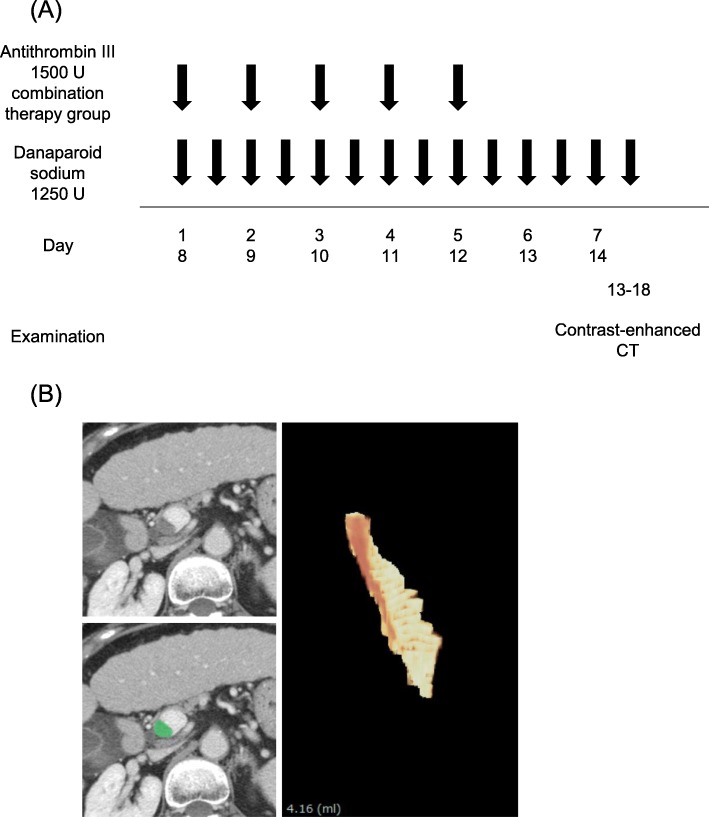
Protocol of danaparoid sodium-based anticoagulation therapy. **a** Patients received intravenous injection of 1250 units of danaparoid sodium twice daily for 2 weeks. Patients belonging to the combination therapy group received additional infusion of AT-III at 1500 units/day from day 1 to day 5 and from day 8 to day 12. PVT was evaluated by using contrast-enhanced computed tomography at the end of 2 weeks of treatment (between days 13 and 18). **b** Measurement of PVT volume. The thrombus was traced on an axial CECT image and the volume of the thrombus was calculated using a 3-dimensional image analysis system (Synapse Vincent Ver. 3 and Ver. 5; Fujifilm Medical Co., Tokyo, Japan)

All patients with PVT included in this study received an intravenous injection of 1250 units of danaparoid sodium (Orgaran; MSD, Tokyo, Japan) twice daily for 2 weeks. Patients belonging to the combination therapy group received an additional drip infusion of AT-III (Nonthron; Nihon Pharmaceutical, Tokyo, Japan) at a dose of 1500 units/day from day 1 to day 5 and from day 8 to day 12 (Fig. 1a).

### Evaluation of PVT

All patients underwent contrast-enhanced computed tomography (CECT) to evaluate for the presence of PVT. We traced the thrombus on an axial CECT image and calculated the volume of the thrombus by using a 3-dimensional image analysis system (Synapse Vincent Ver. 3 and Ver. 5; Fujifilm Medical Co., Tokyo, Japan). Effectiveness was evaluated by CECT between days 13 and 18. Measurement was confirmed by a radiology technologist and the attending physician. PVT volume reduction rate was based on the following calculation:

PVT reduction rate = {(PVT volume before treatment − volume after treatment) / (PVT volume before treatment)} × 100 (Fig. 1b).

### Data collection

We reviewed patients’ medical records and collected demographic, clinical, and laboratory data, including age, sex, hepatitis virus status, hepatic reserve, and imaging data. The Institutional Review Board of Kanazawa University Hospital approved the study’s treatment strategy and study protocol and all patients provided written informed consent for inclusion in the study (No. 2016–096). The study was conducted in accordance with the Declaration of Helsinki.

### Statistical analysis

Statistical analysis was performed with GraphPad Prism software 6.0 (GraphPad Software, San Diego, CA). Categorical variables were compared using the χ^2^-test when appropriate. Student’s t-test was used for continuous variables. Survival rates were analyzed using the Kaplan-Meier method with the log-rank test. All *P* values were two-tailed, and *P* < 0.05 was considered statistically significant.

## Results

### Safety and effectiveness of danaparoid sodium treatment for PVT and effects on the fibrinolytic system

**Fig. 2:**
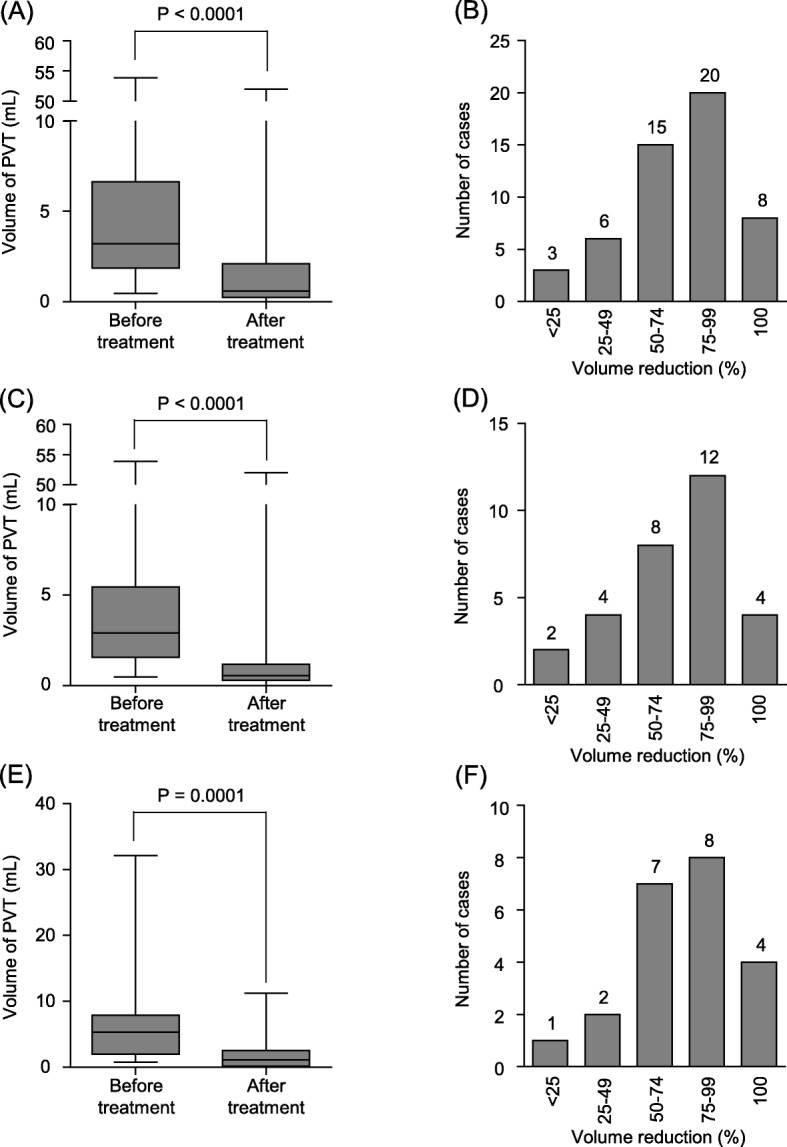
Effectiveness of danaparoid sodium-based anticoagulation therapy. **a** Reduction in the post-treatment volume of the thrombus compared with the pre-treatment volume in all patients. PVT volume significantly decreased from 6.1 ± 8.9 mL to 2.5 ± 7.4 mL (*P* < 0.0001). **b** Distribution of volume change in all patients. **c** Reduction in the post-treatment volume of the thrombus compared with the pre-treatment volume in monotherapy group. PVT volume significantly decreased from 5.1 ± 10.1 mL to 2.9 ± 9.5 mL (*P* < 0.0001). **d** Distribution of volume change in the monotherapy group. **e** Reduction in the post-treatment volume of the thrombus compared with the pre-treatment volume in the combination therapy group. PVT volume significantly decreased from 6.7 ± 7.2 mL to 2.2 ± 3.1 mL (*P* = 0.0001). **f** Distribution of volume change in the combination therapy group

In this study, the primary outcome measures were reduction in PVT volume and safety of the therapeutic intervention. Regarding thrombus volume, after 2 weeks of danaparoid sodium-based anticoagulation therapy, all patients showed considerable reduction in the post-treatment volume of the thrombus compared with the pretreatment volume. PVT volume was significantly decreased from 6.1 ± 8.9 mL to 2.5 ± 7.4 mL (*P* < 0.0001, Fig. 2a). The mean reduction rate of PVT volume was 72%; the distribution of reduction rate of PVT is shown in Fig. 2b.

We considered combination therapy and monotherapy separately. For monotherapy, PVT volume decreased significantly from 5.1 ± 10.1 mL to 2.9 ± 9.5 mL (*P* < 0.0001, Fig. 2c), and the distribution of reduction rate of PVT is shown in Fig. 2d. For combination therapy, PVT volume decreased significantly from 6.7 ± 7.2 mL to 2.2 ± 3.1 mL (*P* = 0.0001, Fig. 2e), and the distribution of reduction rate of PVT is shown in Fig. 2f.

### Effect on the fibrinolytic system and complications

**Fig. 3:**
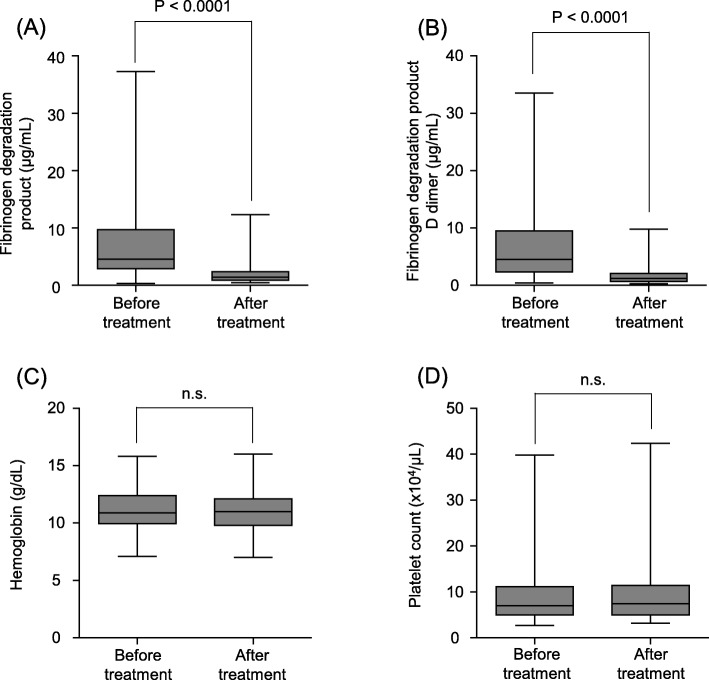
Effect on the fibrinolytic system and time course of hemoglobin levels and platelet counts. **a** FDP changed from 15.7 ± 17.1 μg/mL before treatment to 3.9 ± 3.8 μg/mL at the end of treatment. The FDP level was significantly decreased (*P* < 0.0001). **b** FDP-DD changed from 7.5 ± 8.1 μg/mL before treatment to 1.7 ± 1.6 μg/mL at the end of treatment. The FDP-DD level was significantly decreased (*P* < 0.0001). **c** Hemoglobin levels changed from 11.3 ± 1.8 g/dL before treatment to 11.2 ± 1.9 g/dL at the end of treatment. The hemoglobin level showed no significant difference. **d** Platelet counts changed from 9.0 ± 6.4 × 10^4^/μL before treatment to 9.3 ± 6.4 × 10^4^/μL at the end of treatment, and showed no significant difference

Regarding safety of the therapeutic intervention, we evaluated fibrinolytic system function using fibrinogen degradation products (FDP) (*n* = 50), and fibrinogen degradation products-D dimer (FDP-DD) (n = 50). Patients for whom these values were unavailable were excluded from this analysis. FDP changed from 15.7 ± 17.1 μg/mL to 3.9 ± 3.8 μg/mL (*P* < 0.0001, Fig. 3a) and FDP-DD changed from 7.5 ± 8.1 μg/mL to 1.7 ± 1.6 μg/mL (P < 0.0001, Fig. 3b); the levels decreased significantly at the end of the treatment.

In this study, we defined major bleeding as clinically overt bleeding associated with any of the following: fatal outcome; involvement of a critical anatomic site (intracranial, spinal, ocular, pericardial, articular, retroperitoneal, or intramuscular with compartment syndrome); fall in hemoglobin level > 2 g/dL; or requiring transfusion of > 2 U of whole blood; or packed red blood cells [19]. All patients completed the treatment without the occurrence of major bleeding, thrombocytopenia, or liver dysfunction. The mean hemoglobin level during treatment was 11.3 ± 1.8 g/dL before treatment and 11.2 ± 1.9 g/dL at the end of treatment (Fig. 3c).

Platelet count during treatment was 9.0 ± 6.4 × 10^4^/μL before treatment and 9.3 ± 6.4 × 10^4^/μL at the end of treatment (Fig. 3d). No significant differences were noted in hemoglobin levels and platelet count during the treatment period.

### Factors associated with a 75% reduction of portal vein thrombosis volume

The mean reduction rate of PVT volume was 72%, we determined therapeutic effectiveness cases (effective group) as a reduction of ≥75% in the volume of the thrombus by volume as compared to the pre-treatment volume. Treatment was considered ineffective (ineffective group) if the reduction in the thrombus volume was < 75%.

**Fig. 4:**
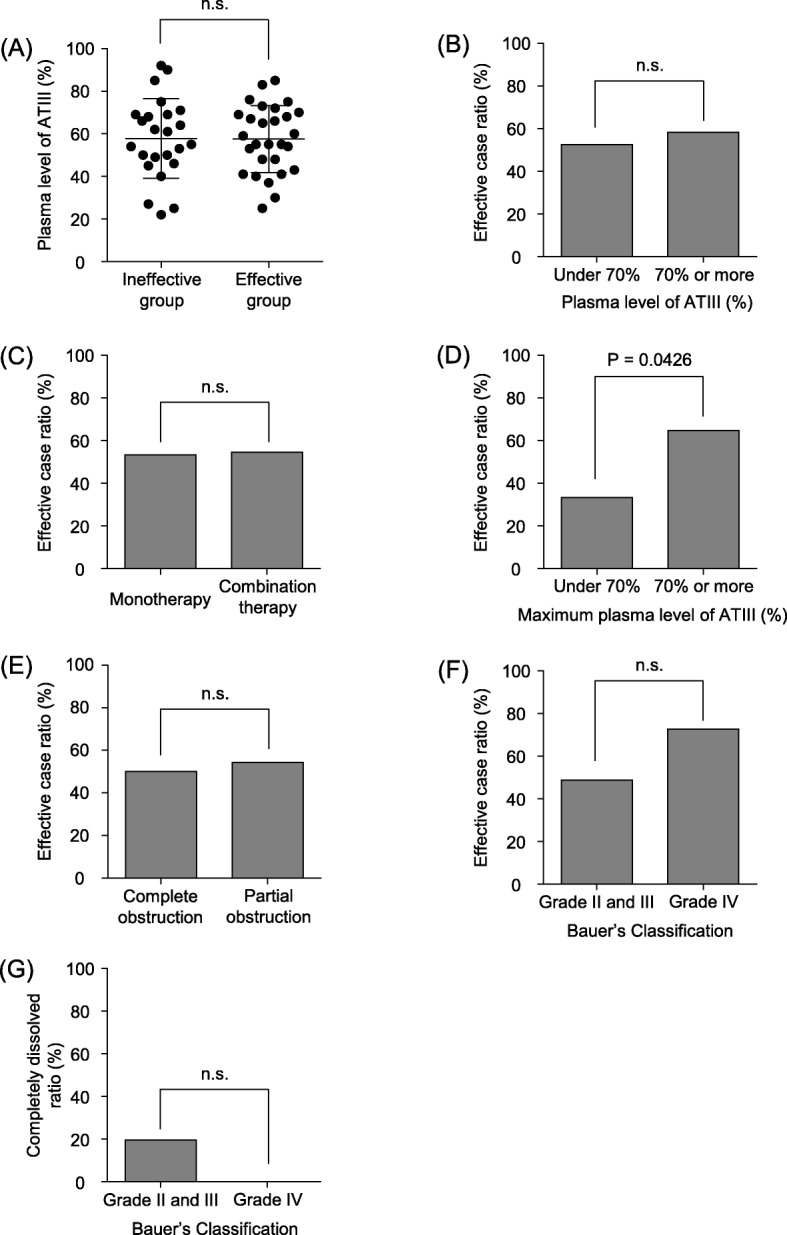
Factors affecting 75% reduction of portal vein thrombosis volume. **a** Plasma AT-III level was 59.5 ± 1.9% and 60.4 ± 14.8% in the ineffective and effective groups, respectively, with no significant difference. **b** No significant difference is seen regarding whether pretreatment AT-III level was within normal range or not in both groups. **c** No significant difference is seen regarding use of AT-III or not. **d** Significantly more cases where treatment was effective for AT-III level > 70% during the treatment period (*P* = 0.0426). **e** No significant difference is seen between partial or complete portal vein occlusion. **f** No significant difference is seen in comparisons between Bauer’s Classification grade II and III patients and grade IV patients. **g** Although the difference was not significant there were 8 patients (19.5%) with completely dissolved thrombus among Bauer’s Classification II and III patients but none among the Bauer’s Classification IV patients

We investigated factors associated with a ≥ 75% reduction of PVT volume. Plasma AT-III level was 57.8 ± 18.7% in the ineffective group and 58.0 ± 16.6% in the effective group, and there was no significant difference (Fig. 4a). Normal plasma AT-III level was defined as > 70%. We compared whether pretreatment AT-III levels were within normal range in the ineffective and effective groups; no significant difference was noted (Fig. 4b). There was also no significant difference in use of AT-III (Fig. 4c). We examined plasma AT-III level measured during the treatment period. There were significantly more cases where treatment was effective for AT-III levels > 70% during the treatment period (*P* = 0.0426, Fig. 4d). For patients with plasma AT-III level ≤ 70% before treatment, it was considered important to correct to normal plasma AT-III level by using AT-III concurrently.

We investigated the relationship between portal vein occlusion rate and therapeutic effect. There was no significant difference between partial or complete portal vein occlusion (Fig. 4e). We compared patients with Bauer’s Classification grades II and III with grade IV patients and found no significant difference (Fig. 4f). Although the difference was not significant, there were 8 cases (19.5%) of complete dissolution of thrombus among the Bauer’s Classification II and III patients but none among Bauer’s Classification IV patients (Fig. 4g).

### Prognosis of portal vein thrombosis patients

**Fig. 5:**
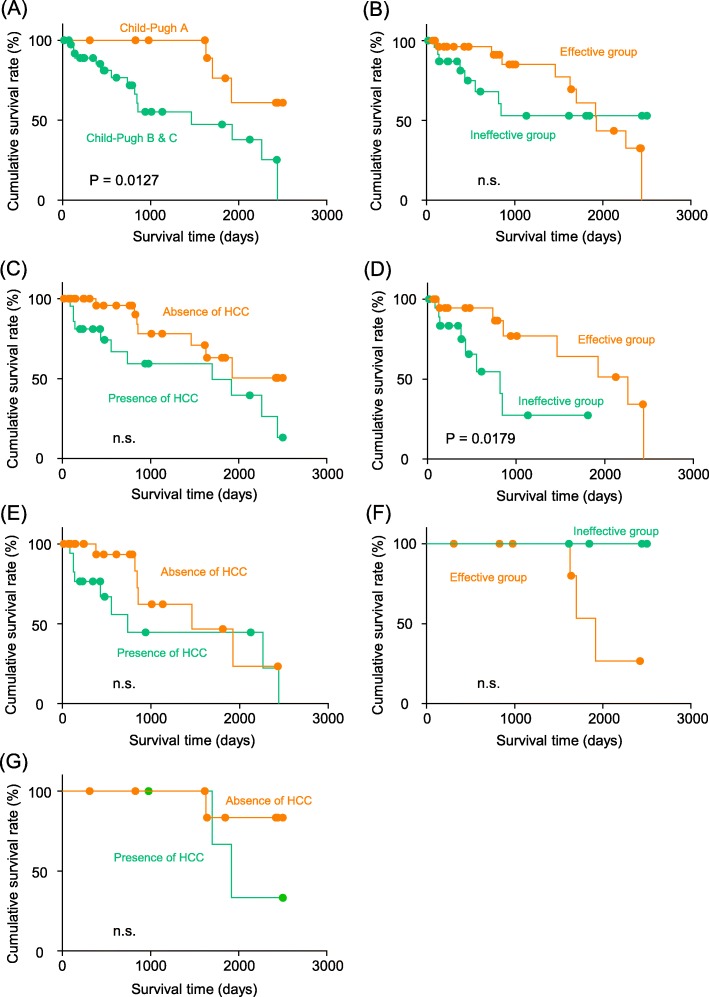
Prognosis of cirrhosis patients with portal vein thrombosis. **a** Compared by hepatic reserve capacity: Compensated cirrhosis (Child-Pugh A, *n* = 13) patients show significantly better prognosis than decompensated cirrhosis (Child-Pugh B and C, *n* = 39) patients (*P* = 0.0127). **b** Compared by treatment effect: No significant difference is seen between the effective group (*n* = 28) and ineffective groups (*n* = 24) (*P* = 0.7128). **c** Compared by presence of HCC: No significant difference is seen between presence of HCC (*n* = 21) and absence of HCC (*n* = 31) (*P* = 0.0618). **d** Compared by treatment effect in Child-Pugh B and C decompensated cirrhosis: Effective group (*n* = 20) shows significantly better prognosis than ineffective group (*n* = 19) (*P* = 0.0179). **e** Compared by presence of HCC in Child-Pugh B and C decompensated cirrhosis: These is no significant difference between presence of HCC (*n* = 17) and absence of HCC (*n* = 22) (*P* = 0.2475). **f** Compared by treatment effect in Child-Pugh A compensated cirrhosis: These is no significant difference between the effective group (*n* = 8) and ineffective groups (*n* = 5) (*P* = 0.0589). **g** Compared by presence of HCC in Child-Pugh A compensated cirrhosis: no significant difference is seen between presence of HCC (*n* = 4) and absence of HCC (*n* = 9) (*P* = 0.3189)

Although the sample size was small, we attempted to investigate prognosis in PVT patients as the secondary outcome measure. We examined hepatic reserve capacity (Compensated cirrhosis (Child-Pugh A) or decompensated cirrhosis (Child-Pugh B or C)), presence of HCC, and treatment effects of danaparoid-based anticoagulation therapy. Hepatic reserve capacity affected the prognosis in PVT patients; Child-Pugh A patients had significantly better prognosis than Child-Pugh B and C patients (*P* = 0.0127, Fig. 5a). Treatment effects (*P* = 0.7128, Fig. 5b) and presence of HCC (*P* = 0.0618, Fig. 5c) did not affect prognosis in this cohort. Thus, prognosis in PVT cases depends on hepatic reserve capacity.

Because of the poor prognosis in decompensated cirrhosis, we examined prognosis for only cases with Child-Pugh B and C decompensated cirrhosis. Treatment effects of PVT affected prognosis of PVT in Child-Pugh B and C cases, and the treatment effective group had better prognosis than the ineffective group (*P* = 0.0179, hazard ratio: 0.22, Fig. 5d). The presence of HCC did not affect prognosis in this cohort (*P* = 0.2475, Fig. 5e). Table 4 shows a comparison between clinical characteristics of Child-Pugh B and C patients in the treatment effective group (*n* = 20) and ineffective group (*n* = 19). There was no difference in the ineffective group. On the other hand, in Child-Pugh A compensated cirrhosis patients, the effects of treatment of PVT (*P* = 0.0589, Fig. 5f) and the presence of HCC (*P* = 0.3189, Fig. 5g) did not affect prognosis.

**Table 4:** Characteristics of Child-Pugh B and C decompensated cirrhosis patients

	Effective group (*n* = 20)	Ineffective group (*n* = 19)	P value
Sex (male / female)	18/2	13/6	n.s.
Mean age (years)	66 ± 8	67 ± 10	n.s.
Etiology (HBV / HCV / HBV + HCV / NBNC)	2/11/1/4	2/9/0/8	n.s.
Child-Pugh score (B / C)	12/8	13/6	n.s.
Hepatocellular carcinoma (absent / present)	10/10	12/7	n.s.
UICC stage of hepatocellular carcinoma			
Absent (none / after curative treatment)	5/5	8/4	
Present (Stage I / II / III / IV)	1/8/0/1	4/3/0/0	
Esophageal varices (absent / present)	0/20	1/18	n.s.
Monotherapy / Combination therapy	8/12	11/8	n.s.
White blood cell count (/μL)	4177 ± 2631	3352 ± 1218	n.s.
Hemoglobin (g/dL)	11.2 ± 1.5	10.8 ± 2.0	n.s.
Platelet count (×10^4^/μL)	9.5 ± 7.7	7.2 ± 3.7	n.s.
Albumin (mg/dL)	2.9 ± 0.3	3.0 ± 0.4	n.s.
Total bilirubin (mg/dL)	1.9 ± 1.2	1.8 ± 1.2	n.s.
Aspartate aminotransferase (IU/L)	54 ± 35	44 ± 14	n.s.
Alanine aminotransferase (IU/L)	36 ± 28	29 ± 13	n.s.
Gamma-glutamyl transpeptidase (IU/L)	52 ± 58	51 ± 77	n.s.
Prothrombin time (%)	60 ± 10	63 ± 15	n.s.
Prothrombin time/International normalized ratio	1.3 ± 0.2	1.3 ± 0.2	n.s.
Fibrinogen degradation product (μg/mL)	16.5 ± 19.7	16.8 ± 11.6	n.s.
D-dimer (μg/mL)	8.4 ± 9.7	7.7 ± 5.7	n.s.
Antithrombin III (%)	53 ± 16	55 ± 19	n.s.

*NBNC* non-B, non-C

These results suggested the possibility that a ≥ 75% reduction of PVT volume may improve prognosis in Child-Pugh B and C decompensated cirrhosis patients with PVT, and danaparoid sodium-based anticoagulation therapy should be considered for such patients. With a hazard ratio of 0.22, the ratio of each group would be 1:1, the event rate would be 38%, and the number of cases would be 39. With a two-sided 5%-level test, the power was 82% and so we consider this test result to be reliable.

## Discussion

In the present study, we assessed the effectiveness of danaparoid sodium-based anticoagulation therapy for PVT in patients with cirrhosis. This study demonstrated that all the patients who received danaparoid sodium-based anticoagulation therapy showed reduction of PVT volume. Recent studies have reported that low-molecular-weight heparin and vitamin K antagonists constitute the two main types of anticoagulants for the treatment of PVT, with a portal vein recanalization rate of 42–100% [9, 20–25]; and meta-analysis showed a portal recanalization rate of 66.6% [26]. In this study, all patients showed reduction of PVT volume following treatment. Most of the cases in this cohort were in the acute stage without portal cavernoma, and this may explain the good treatment outcomes. Another report measured PVT reduction by volume and the reduction rate was 100% [9], but the method for evaluating treatment effectiveness in thrombosis may have differed among past papers. Unification of the treatment evaluation method seems necessary for future advances in PVT treatment. Thus, danaparoid sodium-based anticoagulation therapy may be an equally or more effective treatment as compared with low-molecular-weight heparin and vitamin K antagonists.

Considering the high risk of bleeding due to the reduced synthesis of coagulation factors and presence of esophageal and gastric varices, portal hypertensive gastropathy, and gastric antral vascular ectasia in cirrhotic patients, danaparoid sodium may offer greater safety compared with other anticoagulation agents. In fact, no patients experienced bleeding events in this cohort. Cirrhotic patients generally have low platelet counts due to hypersplenism, and it is well known that using heparin can cause heparin-induced thrombocytopenia. However, in the present study of danaparoid sodium-based anticoagulation therapy, thrombocytopenia was not observed in any of the patients.

A disadvantage of danaparoid sodium-based anticoagulation therapy is that patients require frequent hospital visits or hospitalization because danaparoid sodium is administered as an intravenous injection. Recently, new oral anticoagulation agents that target factor Xa, such as apixaban, rivaroxaban, and edoxaban, have become available [27–29], and these may emerge as novel treatment options for PVT.

In this cohort, after danaparoid sodium treatment, 20 patients were given anticoagulant therapy using vitamin K antagonists and 11 patients were treated with edoxaban. A recent report described the usefulness of maintenance treatment with edoxaban [10]. There are no established criteria for the use of anticoagulant therapy and drug selection after treatment in this cohort, and so further studies on the effectiveness of maintenance treatment with such cases will be needed.

However, there were 21 cases that had not received maintenance treatment. In 1 of these cases, the residual PVT after treatment disappeared after 2 months. A detailed study of transient PVT was recently reported [30]. The PVT in this 1 case may have been transient PVT and may have resolved without anticoagulant therapy. Further study will be needed regarding how to identify which cases of PVT require treatment.

To achieve a ≥ 75% reduction of PVT volume, it is considered important that a plasma AT-III level ≤ 70% be corrected to a normal level by administering AT-III concurrently. AT-III (Nonthron) at a dose of 1500 units is an expensive drug that costs 59,753 JPY per 1500 units. Therefore, we propose that it be used in patients with AT-III of ≤70% and not in all cases, and recommend that additional administration be evaluated by monitoring the AT-III level.

Although this cohort included many cases complicated by HCC, this study showed that treatment of PVT using danaparoid sodium improved prognosis in Child-Pugh B and C decompensated cirrhosis if PVT volume reduced by ≥75% compared with the pre-treatment volume. However, this cohort was small, and regarding the prognosis, further studies are needed.

## Conclusions

Danaparoid sodium-based anticoagulation therapy was effective and safe for PVT in patients with cirrhosis. Return of plasma AT-III level to normal range during the treatment period contributes to a ≥ 75% reduction of PVT volume. The prognosis in PVT patients depends on hepatic reserve capacity. Anticoagulation therapy for PVT by danaparoid sodium in Child-Pugh B and C decompensated cirrhosis patients, a ≥ 75% reduction of PVT volume may improve the prognosis.

## Data Availability

The datasets used and/or analyzed during the study are available from the corresponding author on reasonable request.

## Abbreviations

AT-III: Antithrombin III
CECT: Contrast-enhanced computed tomography
FDP: Fibrinogen degradation products
FDP-DD: Fibrinogen degradation products-D dimer
HCC: Hepatocellular carcinoma
LPV: Left branch of the portal vein
PVT: Portal vein thrombosis
RPV: Right branch of portal vein
SMV: Superior mesenteric vein
